# Widow cleansing and inheritance among the Luo in Kenya: the need for additional women-centred HIV prevention options

**DOI:** 10.7448/IAS.17.1.19010

**Published:** 2014-06-26

**Authors:** Brian Perry, Lennah Oluoch, Kawango Agot, Jamilah Taylor, Jacob Onyango, Lilian Ouma, Caroline Otieno, Christina Wong, Amy Corneli

**Affiliations:** 1 Social and Behavioral Health Sciences Department, FHI 360, Durham, NC, USA; 2 Impact Research and Development Organization, Kisumu, Kenya

**Keywords:** Kenya, Luo, widow inheritance, widow cleansing, condom, HIV prevention, FEM-PrEP

## Abstract

**Introduction:**

The customs of widow cleansing and widow inheritance are practiced in several communities throughout sub-Saharan Africa. In the Nyanza Province of Kenya, according to tradition, Luo widows are expected to engage in sexual intercourse with a “cleanser,” without the use of a condom, in order to remove the impurity ascribed to her after her husband's death. Luo couples, including widows, are also expected to engage in sex preceding specific agricultural activities, building homes, funerals, weddings, and other significant cultural and social events. Widows who are inherited for the purpose of fulfilling cultural obligation have a higher prevalence of HIV than those who remain un-inherited or are inherited for the purpose of companionship.

**Methods:**

As part of a larger descriptive qualitative study to inform study procedures for FEM-PrEP, an HIV prevention pre-exposure prophylaxis clinical trial, we conducted 15 semi-structured interviews (SSIs) with widows, 15 SSIs with inheritors, and four focus group discussions with widows in the Bondo and Rarieda districts in Nyanza Province to explore the HIV risk context within widow cleansing and inheritance practices. Thematic qualitative analysis was used to analyze the data.

**Results:**

The majority of widows reported in the demographic questionnaire being inherited, and most widows in the SSIs described participating in the cleansing ritual. We identified two main themes related to HIV prevention within the context of widow cleansing and inheritance: 1) widows must balance limiting their risk for HIV infection with meeting cultural expectations and ensuring that their livelihood needs are met, and 2) sexual abstinence undermines cultural expectations in widowhood while the use of condoms is deemed inappropriate in fulfilling culturally prescribed sexual rituals, and is often beyond the widow's ability to negotiate.

**Conclusions:**

Women-controlled HIV prevention methods such as antiretroviral-based oral pre-exposure prophylaxis, vaginal gels, and vaginal rings are needed for HIV-negative widows who engage in sexual rituals related to widowhood.

## Introduction

Widow cleansing and widow inheritance are cultural practices observed in many communities in sub-Saharan Africa [1–7]. Among the Luo community in Kenya, according to tradition, after a woman's husband dies, she must engage in sexual intercourse without a condom with a “cleanser,” often a non-relative of the deceased husband, to remove the impurity she is believed to have acquired from the death of her husband [8]. After a widow has been “cleansed,” she is expected to be inherited by a man, traditionally an in-law. However, previous studies have reported that in-laws are becoming less willing to inherit the widows of their relatives because of the economic burden of supporting the widow and her family as well as the risk for acquiring HIV from the widow [9–13]. This has led some men (non-relatives) to commercialize the practice. Professional inheritors, who serially or concurrently inherit widows from different communities for money or other forms of payment, are becoming more frequent as the demand for willing inheritors grows [9,10,13].

Inheritance is often considered distinct from marriage. Inheritance relationships are based on prescribed roles rather than the commitment and permanent bonds of marriage. Widows remain “married” to their husband even after their passing [10,13–15]. If an inheritance relationship ends, there is no such bond or claim held by the inheritor. Inheritors are simply “filling in” or playing the role of a “husband.” Inheritors are expected to help the widow comply with sexual rituals and, to a varying degree, provide the widow financial and emotional support. Particularly in the case of professional inheritance, the widow and inheritor will come to a mutual understanding of the scope of the relationship and if a professional inheritor is perceived to exceed or not fulfil the role expected of him, the relationship can simply dissolve [12,13].

Aside from the initial cleansing ritual, widows, as well as married women, are expected to observe other sexual norms common in the Luo community. For example, they may be expected to engage in sexual intercourse during the establishment of a home; during agricultural cycles such as tilling the land, planting, and harvesting; and when participating in the funeral or marriage ceremonies of some relatives [4,9. 10,16,17]. In each of these cases, a widow must find a sexual partner to help her fulfil these required rites. Fulfilling these sexual rituals often means engaging in sex without a condom, because the practices are considered incomplete unless semen and vaginal fluids mix during sexual intercourse [1,10,11,16]. While an increasing number of widows voluntarily choose not to observe the traditions, many still engage in these practices to conform to societal norms or because they are compelled to do so by their husbands’ families, their own families, or the belief that engaging in the tradition will ensure that they or their children will not be ostracized or face illness or other misfortunes [3,9,10].

Widow cleansing and widow inheritance continue to be practiced in the Nyanza Province of Kenya, which was one of the locations of FEM-PrEP, a Phase III HIV prevention pre-exposure prophylaxis (PrEP) clinical trial [18]. Agot *et al*. [1] have shown that the Nyanza Province has a high prevalence of HIV infection among widows (63%), and that widows who are inherited for the purpose of performing sexual rituals have increased odds of being infected with HIV when compared with widows who are not inherited or are inherited for the purpose of companionship [1]. Limited condom use among widows living in this area was also documented in this study, with only 2.7% of widows reported having used a condom since the death of their husbands [1].

In this paper, we describe the sexual risk-taking behaviours of Luo widows and inheritors to illustrate the need for women-centred HIV prevention methods, such as antiretroviral-based (ARV-based) oral PrEP, vaginal gel, and vaginal ring.

## Methods

### Data collection

We conducted 15 semi-structured interviews (SSIs) with widows, 15 SSIs with inheritors, and four focus group discussions (FGDs) with a total of 37 widows (distinct from those who participated in the SSIs) in the Bondo and Rarieda districts in Nyanza Province. The final sample sizes were based on the degree of information saturation (i.e. the point at which there was no new information or themes observed in each of the participant groups) assessed during data collection. Previous research has shown that saturation can occur within the first 12 interviews conducted in a relatively homogeneous group when the objective of the research is to understand common perceptions and experiences [19]. The SSIs and FGDs were conducted between October 2007 and January 2008 as part of a larger qualitative descriptive study to inform study procedures for the FEM-PrEP trial [18] prior to its implementation. Widows were purposefully selected [20] based on their age (must have been between 18 and 35 years old) and sexual activity (must have had, on average, at least one sex act per month in the past three months); widows were not selected based on their inheritance status. Inheritors were selected if they had formally or informally served as a “cleanser”/inheritor to a widow in the past year and not based on their relation to their inherited partner (relative vs. non-relative). Widows were primarily recruited through widows’ groups and inheritors were recruited through key informants in the community. We also collected demographic data, such as age, marital status, occupation, education, and inheritance status for each of the SSI and FGD participants.

We chose to include widows and inheritors in our preparedness research because we knew that several participants in the subsequent FEM-PrEP trial would likely be widows who engage in rituals surrounding widowhood and we believed we needed to have a better understanding of their HIV risk within this context in order to inform FEM-PrEP recruitment and study procedures, such as risk reduction counselling. Therefore, in the SSIs with widows, we asked participants to describe their individual beliefs in their risk of acquiring HIV as well as behaviours, such as whether or not they ever used condoms with inheritors, the context in which condoms are used or not used with inheritors, and if condom use differs between types of inheritors (i.e. late-husband's relative versus non-relative). We asked inheritors to describe periods of increased activity in their role as inheritor, as well as situations in which they use and do not use condoms in their role as inheritor, particularly in situations where they have an ongoing relationship with a widow. We also asked about concurrent sexual partnerships and condom use with concurrent partners. Because data from this study were to inform FEM-PrEP study procedures, we also asked participants questions related to perceptions of research and acceptability of daily pill taking within the context of a clinical trial (data not presented here).

We used a vignette approach to explore normative views surrounding widow inheritance in the FGDs [21]. Participants were asked to describe situations surrounding condom use for a 26-year-old fictional character whose husband died a year ago and who goes to a professional inheritor for the purpose of observing the rituals in widowhood. FGD participants were also asked to describe condom use scenarios for another similar fictional character that was inherited by her brother-in-law after her husband died.

The SSIs and FGDs were conducted in Dholuo (the local language) and by interviewers or moderators of the same ethnic community and gender as the participants. All SSIs and FGDs were audio-recorded. The recordings were simultaneously transcribed and translated into English following a transcription protocol [22]. The study was approved by the Kenyatta National Hospital-University of Nairobi Ethics and Research Committee in Kenya and the Protection of Human Subjects Committee at FHI 360 in the United States.

### Data analysis

We used qualitative thematic analysis techniques to analyze the data. All 34 SSI and FGD transcripts were read in their entirety and structurally coded, based on questions in the interview guide, by two analysts using qualitative data analysis software [23]. Structural coding reports pertaining to inheritance relationship dynamics and condom use were analyzed using data-driven thematic analysis techniques [24]. Three qualitative analysts identified salient content areas from the structural coding reports as they emerged in the transcripts and developed relevant content codes to capture these topics. Two of the analysts reviewed all transcripts together, and content codes were then applied to the text using NVivo 9. Once content coding was complete, coding reports were reviewed to identify potential themes. Two different analysts (including the lead author) verified these themes and further solidified underlying concepts related to general sexual behaviours in inheritance relationships. Final themes were grouped based on how participants described their risk for HIV infection and their perceived ability to prevent infection given the cultural expectations surrounding widow cleansing and inheritance and the availability of appropriate prevention options.

## Results

We identified two main themes related to HIV prevention within the context of widow cleansing and inheritance: 1) widows acknowledge that they face greater risk for HIV by engaging in cleansing and inheritance rituals, particularly with professional inheritors, but that they must balance this risk with cultural expectations and ensuring their livelihood needs are met, and 2) current HIV prevention methods, such as abstinence and condoms, are considered inappropriate or beyond the widow's ability to negotiate.

### Demographic characteristics

Widows in the SSIs ranged from 23 to 35 years of age. Most had a primary school education or less and were employed full time as market vendors or selling fish (Table 1). The majority reported that a non-relative had inherited them and more than half of the widows reported participating in sexual cleansing rituals after the death of their husbands (Table 2).

**Table 1:** Demographic characteristics

	SSIs (*N*=30)		

	Widows, *N*=15	Inheritors, *N*=15	FGDs (*N*=4) Widows, *N*=37
Age, mean years (range)	29 (23–35)	42 (19–76)	31 (22–35)
Marital status, *n* (%)			
Never married	–	1 (7)	–
Not married but living with partner	–	1 (7)	–
Married	–	9 (60)	–
Divorced	–	1 (7)	–
Widower	–	3 (20)	–
Inherited (widows only)	12 (80)	–	26 (70)
Not Inherited (widows only)	3 (20)	–	8 (22)
No response	0 (0)	0 (0)	3 (8)
Education, *n* (%)			
Completed secondary school	1 (7)	0 (0)	3 (8)
Some secondary school	2 (13)	4 (27)	7 (19)
Completed primary school	7 (47)	4 (27)	8 (22)
Some primary school	5 (33)	7 (47)	16 (43)
None	0 (0)	0 (0)	1 (3)
No response	0 (0)	0 (0)	2 (6)
Employment, *n* (%)			
Full time	8 (53)	2 (13)	9 (24)
Part time	1 (7)	7 (47)	10 (27)
No	6 (40)	6 (40)	16 (43)
No response	0 (0)	0 (0)	2 (6)

SSI, semi-structured interview; FGD, focus group discussion.

**Table 2:** Sexual cleansing and inheritance among participants of semi-structured interviews (*N*=15)

Cultural practice	*n* (%)
Sexual cleansing	
Took part in traditional cleansing	9 (60)
By non-relative	7 (78)
By a relative	2 (22)
Did not take part in cleansing ritual	6 (40)
Inheritance	
Inherited	12 (80)
By a non-relative	10 (83)
By a relative	2 (17)
Not inherited	3 (20)

Widows in the FGDs were similar to the widows in the SSIs. They were between 22 and 35 years of age and had similar levels of schooling and livelihoods, though a lower percentage was employed full time than those in the SSIs. Most had been inherited by the time of the FGD (Table 1).

Inheritors in the SSIs were aged 19–76 years and had at least some primary school education. Only nine inheritors had either part- or full-time employment, which primarily included working as fishermen or as *bodaboda* [bicycle or motorcycle taxi] drivers. Most reported having multiple concurrent sexual partners. Two-thirds (*n*=9) were married (to a woman other than the one they inherited), three were widowers, and the remaining inheritors were either single or divorced. The distinction between “marriage” and “inheritance” was often highlighted by inheritors referring to their inherited partner as someone “like” their wife.

### Balancing HIV risk with meeting cultural expectations and ensuring livelihood needs are met

Fear of acquiring, or spreading HIV, was often discussed by widows when they talked about widow cleansing and inheritance. Nearly all of the widows in the SSIs, and many in the FGDs, expressed concern that having sex without a condom with an inheritor contributes to the spread of HIV in the community. Several widows stated that particularly in relationships with professional inheritors, an inheritor's “movements” (i.e. sexual behaviours outside of the inheritance relationship) are often unknown and it is uncommon to know his HIV status before entering into the relationship:

Even if I am clean [HIV-negative] right now tomorrow I can get it accidentally … I am at risk because the person that I have is far away [and therefore unable to know her partner's movements] … Also he is someone who, the way I see him, he is someone who I see likes young girls. Now I can be at risk. (Inherited widow, SSI)

I feel that that inheritor – that one that I got along the beach – I feel like he is not straight forward … So sometimes I get worried deep in my heart because he is walking here and there [having multiple partners], I feel that he might bring for me a disease. (Inherited widow, SSI)

Though widows acknowledged that engaging in inheritance relationships place them at risk for HIV infection, both widows and inheritors mentioned that widows need an inheritor to not only help them perform specific sexual rituals throughout the year to ensure their cultural obligations are met but also to help with practical needs, such as building a new home (or make necessary home repairs), plough or sow fields, and harvest crops. However, participants also acknowledged that some inheritors may refuse to offer the degree of support the widow was expecting. Several widows in the SSIs and many in the FGDs spoke about their own experiences or those of other widows in the community whose inheritor refused to perform all of the duties expected of them:

He [her in-law] refused to do some [expected tasks]. He only finished that one of going home and shaving [cleansing ritual] then he refused to change the house [the custom of building a widow a new home after her husband dies]. He said he is not changing; he has not changed for his own home. (Inherited widow, SSI)

Several participants elaborated that this can often have devastating consequences on the widow's well-being. One widow stated that because her inheritor refused to build her a home, she was left homeless:

My house was not in a good condition. I was required to change my house. Now the changing of the house is what brought about quarrels. That led that person [her professional inheritor] to leave; it was not easy for him. Now later, I became discouraged and I stayed in my house for some time. Then last year, 2006, my house collapsed. There was some heavy downpour in November that destroyed my house until it collapsed. Now I do not have a house. (Inherited widow, SSI)

According to widows and inheritors in the SSIs and FGD participants, when widows’ basic needs are not met – whether to help provide livelihood needs or to help perform a sexual ritual – they will often have to find a different inheritor. Given the “contractual” nature of the relationship between a widow and professional inheritors, several widows and inheritors in the SSIs and widows in the FGDs suggested that a widow has the freedom to dismiss a professional inheritor if at any time she feels he is not fulfilling her needs or has otherwise become undesirable:

A widow can tell an inheritor to leave because he is not helping her in any way and she can then look for another inheritor and if he is not helping her, she will ask him to leave and so on. In a year, [the vignette character] can have even ten inheritors … If he is someone who doesn't come from your husband's clan, she just changes them. Because these are the people she has brought, they have not married her she has married them. (Inherited widow, FGD)

Similarly, one inheritor described how he had been “hired” by multiple widows to help them fulfil the sexual rites that their in-laws had refused to perform:

Somebody [an in-law] inherited her then later on people influenced him and discouraged him that it is a taboo so the person left her. So I am the one who went and lit for her fire. I lit the fire for those two days and then left. Meaning, I had sex with her to fulfil the sexual ritual of warming a new house. I slept with her once, [stayed] two days then I left. I welcomed her in the house as a man … This I have done to three women … I just went, I was like a hire. I was just taken by bicycle and went and did that thing [had sex with her] then I just left for good. (Married inheritor, SSI)

As participants in one FGD pointed out, this can lead to widows engaging in serial relationships with inheritors to meet their various needs, further increasing their potential exposure to HIV:

If she finds the man is not willing to help her she will then tell him to leave so that she can find another person who can build for her … So she will keep changing men until she gets the right man and this can bring problems to [the vignette character]. (Inherited widow, FGD)

### Current HIV prevention methods are inappropriate in inheritance relationships

Nearly all of the SSI participants (widows and inheritors) discussed how inheritance relationships are based on the obligation to fulfil societal expectations and gender roles, both throughout the year and at specific moments in one's life. Sex was frequently discussed and considered an essential component of fulfilling these roles. One 60-year-old, married inheritor stated that “through sex they [widows and their families] become free [interviewer note: from impurities conferred to her by her husband's death]” to build new homes, till and sow fields, or harvest crops and subsequently allow their children or in-laws to do the same. As a result, abstinence among widows was rarely discussed, and only three widows mentioned remaining abstinent since their husbands’ deaths. One of these women was abstinent because she was observing the culturally prescribed mourning period known as *sawo*. Another implied that, based on cultural taboos, her children were still too young for her to seek an inheritor. Both of these women mentioned that because of cultural expectations and fear of being ridiculed by family, they would eventually find an inheritor, though they expressed concern that doing so may contribute to the spread or acquisition of HIV. Only one widow said that she planned to remain un-inherited because of her fear of acquiring HIV from an inheritor.

Both inheritors and widows described that sex without a condom is an expected norm when engaging in intercourse for the purpose of fulfilling culturally prescribed sexual rites. As described by participants from both SSIs and FGDs, it is particularly important during the cleansing ritual because sperm and vaginal fluids must mix in order for cleansing to be fulfilled:

Normally during the cleansing ritual they don't consider using a condom because using the condom is not like somebody has cleansed … Cleansing is accomplished when those things [sperm] have entered a woman. So when those things [sperm] are removed out, and are poured out, it is like cleansing is accomplished half way. (Married inheritor, SSI)

If you use condoms that means you have broken the customs of inheritance. Now you have to have sex without. (Inherited widow, FGD)

Several widows and inheritors participating in the SSIs reported that inconsistent condom use was common. Although nearly all the widows perceived that they were at risk for acquiring HIV from their inheritors, only a few stated that they are able to negotiate condom use with their sexual partners on a consistent basis. Nine widows and five inheritors stated they had previously used condoms with their partners in inheritance relationships, but use was often dependent on the partners or on specific circumstances and was inconsistent. Among the widows who reported condom use, condoms were generally used in new or casual inheritance relationships to prevent disease, and then only when agreed upon by both partners. Participants described that the ability to negotiate condom use may also vary depending on the inheritance relationship. Though in-laws were generally perceived to be less risky than non-relatives, FGD participants suggested that widows may have less autonomy in their relationship with in-laws and potentially lower ability to negotiate condom use with these partners:

You know, when you go to your brother-in-law, you go to his house and discuss [interviewer note: the possibility of him inheriting you], then when he comes in the house, you again tell him that we must have sex using condoms. He is someone who has never been tested, he must be suspicious, that, “why is this woman telling me this?” He might end up going back without inheriting you. (Widow [inheritance status unknown], FGD)

Among widows participating in the SSIs, common reasons for not using condoms with inheritors were refusal by the inheritor, to avoid suspicion of having HIV, the belief that both partners were HIV-negative, the need to fulfil the custom of cleansing, and to become pregnant. Most inheritors described that condoms were most commonly used at the beginning of any new relationship to prevent disease and if the partner (wife or widow) was menstruating or was pregnant. Condom use, however, was unlikely among inheritors when they were performing the cleansing ritual with widows, when in long-term relationships, and with partners over a certain age (as older women were perceived to be less likely to have HIV). Many inheritors also described that condom use was infrequent when men wished to avoid stirring distrust in the relationship or to allow for procreation.

Widows in the FGDs expressed mixed opinions as to whether widows can use condoms while fulfilling their other sexual obligations. A few FGD participants suggested that the use of condoms could potentially be negotiated during other sexual encounters with inherited partners after the initial cleansing has been performed:

When you have done it for the first time after you have been inherited then you can just use condoms but on the first day, you do not use condoms. (Inherited widow, FGD)

This claim was also expressed by most of the inherited widows in the SSIs, who stated that they have used condoms with their inherited partners at some time in their relationships. However, most of the FGD participants thought that inheritors prefer not to wear condoms and that if they refuse, widows have little choice but to oblige:

[For] the professional inheritor, that act of sex is the one which brought him, so if you again come to him that there is a condom here; he knows that when he is using this condom, he is not going to have any feeling while having sex. So he would like it to be plain the way it is. (Un-inherited widow, FGD)

The desire to have children also influenced condom use among some participants in the SSIs. Three inheritors stated that they either have already conceived or would like to conceive children with their inherited partners. Most widows in the SSIs, on the other hand, were primarily ambivalent about conceiving or did not want to conceive with their inherited partners; however, most were also aware that if their inheritors desired children, they had few alternatives (other than finding a new inheritor):

We have been using condoms but for the last one year he changed his mind, because he also wants to have his own child so he doesn't see the need to use condoms … Personally I had said no to have more children because the children my former husband left me with have just been depending on his help but if he is willing to take care, I will have one. (Inherited widow, SSI)

## Discussion

Sex is an essential component of Luo customs and societal expectations. As a result of their expected participation in cleansing and inheritance customs, our findings suggest that widows believe that they are at greater risk for HIV infection. Abstinence and condoms were the only two effective HIV prevention options discussed by study participants and available to widows, although neither option is likely feasible given the cultural expectations of sexual rituals in widowhood. Abstinence can be difficult because of a widow's fear of being ostracized by her family and community if she does not engage in prescribed sexual rituals, and consistent condom use is unlikely because of pressure to appropriately fulfil the rituals, to conceive a child, or because the male partner refused to use condoms.

Despite these findings, our data also suggest that apart from the cleansing ritual, widows may have more autonomy in inheritance relationship with non-relatives compared to in-laws because of the casual nature of the relationships. The often “contractual” arrangements widows make with professional inheritors may allow them greater autonomy to engage in protected intercourse while still observing the cultural rites and desires of their partners. However, women may still have little power regarding condom use in these sexual relationships because the ultimate control of the available HIV prevention option (i.e. condoms) is perceived to reside with the male partner. If the inheritor is unwilling to wear a condom to perform the sexual rituals, the widow often has no choice but to comply or find another willing inheritor.

The findings from our study as well as from other studies suggest that cleansing and inheritance customs provide a social institution by which widows can receive assistance and potential companionship, similar to what they would have received from a husband [15,25,26] but that also legitimizes multiple concurrent sexual partnerships. Inheritors will often be in concurrent relationships with one or multiple wives, as well as one or multiple inherited widows [13,27]. Because of the casualness of these relationships, many widows may go through several professional inheritors and, likewise, many professional inheritors will move from one widow to the next [9,10,12,13]. Each new partnership increases the potential exposure to HIV, which puts not only the widows or inheritors but also other long-term partners (i.e. wives and other widows) at greater risk for infection.

Ultimately, our findings highlight the need for women-centred HIV prevention options such as ARV-based oral PrEP, which has been shown effective at reducing the risk of HIV acquisition among women in serodiscordant relationships [28], as well as vaginal gels [29], and the vaginal ring, of which effectiveness is currently being confirmed (1% tenofovir gel) [30] or evaluated (vaginal ring) [31,32]. These options would presumably provide HIV-negative widows with additional tools they need for reducing their risk for HIV while maintaining their ability to engage in the custom of widow inheritance.

While our primarily content-based analytical approach allowed us to observe recurring themes regarding the barriers and facilitators to condom use among widows and inheritors in our sample, our non-random selection of participants and our small sample size limit the generalizability of these results. Nonetheless, our findings mirror those in previous research on Luo customs, as described above.

## Conclusions

The use of new HIV prevention products must be situated within the cultural and societal contexts in which women at risk for HIV infection live and are potentially exposed to the virus. Within the context of widowhood among the Luo, abstinence and condoms are not practical approaches because sex is essential and because sperm and vaginal fluids must mix to effectively fulfil the cultural rites. Women-centred HIV prevention options such as ARV-based oral PrEP, vaginal gels, and vaginal rings, are needed to reduce the HIV risk among HIV-negative widows. Future research should focus on studying the acceptability of women-centred HIV prevention products among widows and inheritors.
